# Cold Affusion in Remittent Fever

**Published:** 1846-10

**Authors:** 


*Cold affusion in Remittent Fever*—The following is from a review of Prof. Clymer's late work on Fevers, by one of the editors of the St. Louis Med. and Surgical Journal. Neither Prof. Clymer's, nor Prof. Dickson's work, has, as yet, reached this city. We are therefore obliged to make a long arm in reaching a small portion of their valuable contents, through our St. Louis cotemporary.—*Ed.*

*Cold affusion in Remittent Fever.*—"Emetics," he observes, "are now generally abandoned in the treatment of this form of fever, it being found that they aggravate the gastric disease which is usually so annoying." We are sorry that this remark is not true in regard to the treatment of this form of fever in all parts of this country, but unfortunately for the patient it is not—the abominable emetic practice being still adhered to by many, notwithstanding, as our author remarks, "they aggravate the gastric disease which is usually so annoying." General blood-letting he thinks rarely necessary, while he recommends local bleeding by means of cups and leeches, under proper circumstances. In this we fully agree with him. He who has had the least experience in the treatment of fevers of the south and west, will have learned that the lancet, so far from being generally indicated, cannot be used without positive injury to the patient. In lieu of the lancet, and as a most efficient febrifuge, he quotes from the learned work of Dr. Dickson, of Charleston, S. C., what he says on the application of cold water, particularly the cold affusion. As we are great advocates for the free use of cold water, though by no means inclined to hydropathy, we will close our notice with Dr. D.'s remarks on this subject. "All that we can hope to anticipate from blood-letting, may be obtained in a majority of cases by the use of the bath, while the latter possesses this striking and obvious advantage, that we can repeat it as often as the symptoms are renewed that require it. Nor can I help expressing my surprise at the very limited resort of my professional brethren to it, when I consider how instinctively we desire it as a relief from the burning heat that oppresses us, and how certain and immediate a means it is of affording this relief. Of the three modes of employing it, namely, affusion, immersion and ablution, the first is the most impressive and efficacious—the last the least liable to objection or risk in doubtful cases. The particular indications which demand the resort to it, unhesitatingly, are to be found in the youth and general vigor of the patient, and the heat and dryness of the surface. The local determination which it controls most promptly is that to the brain, shown by head-ache, flushed face, red eyes, delirium, &c., with a full, hard, bounding pulse. Seat your patient in a convenient receptacle, and pour over his head and naked body from some elevation, a large stream of cold water: continue this until he is pale and he shivers. On being dried and replaced in bed, a general sense of comfort and refreshment will attest the benefit derived from the process, which, as I have said above, may be repeated whenever the symptoms are renewed, which it is so well adapted to remove. If the shock in the shower-bath or cataract be too great, immersion, which many prefer, may be substituted. Few shrink from this, and almost every one will evince the high gratification and enjoyment derived from it. One of the pleasantest effects following the bath is the complete relaxation of the surface which it so often brings on, attended with a copious and salutary sweat. I need not warn you against the nearly obselete practice of endeavoring to accelerate or increase this by wrapping in blankets, or shutting up the apartment, or warming it artificially. The patient is to be covered agreeably to his sense of comfort; and though I would not place him in a current or draught of air, I would have his chamber fully and freely ventilated. * * * * The affusion of cold water locally upon the head, in a stream of some height, by the spout bath, is of inestimable advantage in cases where the cerebral determination is inordinately violent, dangerous or tenacious, and will have to be repeated far oftener than it would be proper to take the patient out of bed for the administration of the general bath. Support him in a leaning posture over the bed-side, and dash the current from a pitcher over the vortex for some minutes, and from some elevation above him. * * * * The cold bath in its several modes of application is prohibited, let me remind you, when the patient is of feeble habit of body, much advanced in age, much exhausted and enfeebled at the time; when the pulse is weak or the skin cool, or covered with moisture; when the lungs are oppressed or inflamed, and when diarrhœa is present. Its repetition is forbidden when it has occasioned a protracted chill or rigor, or the patient has continued to feel cold or uncomfortable from it." McP.

